# Detection of West Nile virus via retrospective mosquito arbovirus surveillance, United Kingdom, 2025

**DOI:** 10.2807/1560-7917.ES.2025.30.28.2500401

**Published:** 2025-07-17

**Authors:** Robert C Bruce, Anthony J Abbott, Ben P Jones, Bathsheba L Gardner, Estela Gonzalez, Andra-Maria Ionescu, Madhujot Jagdev, Ava Jenkins, Mariana Santos, Katharina Seilern-Macpherson, Hugh J Hanmer, Robert A Robinson, Alexander GC Vaux, Nicholas Johnson, Andrew A Cunningham, Becki Lawson, Jolyon M Medlock, Arran J Folly

**Affiliations:** 1 Animal and Plant Health Agency, Addlestone, United Kingdom; 2 Medical Entomology and Zoonoses Ecology Group, UK Health Security Agency, Salisbury, United Kingdom; 3 Institute of Zoology, Zoological Society of London, London, United Kingdom; 4 British Trust for Ornithology, Thetford, United Kingdom

**Keywords:** *Orthoflavivirus*, *Aedes vexans*, emerging infectious disease, xenosurveillance

## Abstract

In March 2025, as part of ongoing enhanced surveillance for mosquito-borne *Orthoflaviviruses,* West Nile virus (WNV) RNA was detected in two pools of female *Aedes vexans* collected in July 2023 in Nottinghamshire, England. Sequencing and phylogenetic analysis of a 402 bp fragment indicate clustering with WNV lineage 1a. The exact origin of this virus remains unclear, but this finding indicates a historic WNV presence in the United Kingdom. Surveillance has not provided evidence of further WNV transmission to date.

West Nile virus has been detected in mainland Europe since the mid-1990s [1] where two lineages (1a and 2) circulate and cause sporadic outbreaks, with disease recorded in wild birds [2], horses [3] and humans [4]. Although the virus has been recently detected in Germany and the Netherlands [5,6], no evidence of emergence has been previously recorded in the United Kingdom (UK). Here, we describe the first detection of WNV lineage 1a in 2025 from a native mosquito population collected in the UK in 2023.

## *Orthoflavivirus* surveillance in the United Kingdom

Following the emergence and establishment of Usutu virus (USUV, *Orthoflavivirus*) in the UK in 2020 [7], enhanced arbovirus surveillance under the One Health paradigm was established through the VB-RADAR (Vector-borne Real-time Arbovirus Detection And Response) project (https://www.vb-radar.com). This project includes screening mosquitoes from a range of wetland and urban areas deemed at high risk of arboviral introduction [8].

**Figure 1:**
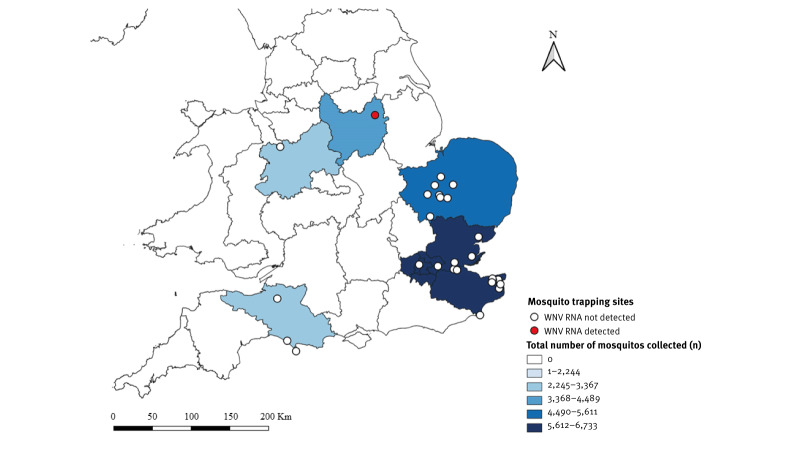
Geographical distribution of mosquito surveillance for arbovirus screening, and confirmation of WNV RNA detection site, United Kingdom, March 2023–March 2025

From March 2023 to March 2025, 31,582 mosquitoes comprising 22 species (including 7,994 *Culex pipiens*, 2,933 *Cx. modestus* and 20,655 of 20 other species) were collected using a range of active trapping methods, targeting both bird- and mammalian-feeding species at 26 sites in the UK (Figure 1) and were analysed for arboviral presence. The full breakdown of mosquito specimens screened for arboviral presence is provided in Supplementary Table S1. Two *Aedes vexans* pools from the same site in Nottinghamshire, England, tested positive for WNV RNA, with the remaining 3,637 pools being negative. 

WNV: West Nile virus.

A map of the United Kingdom showing the number of mosquitos collected for arboviral surveillance, and locations where West Nile virus RNA was either detected or was not detected. The WNV positive pools were collected in Nottinghamshire, England in 2023. Regional separation is illustrated at NUTS2 level (produced using QGIS Maidenhead v3.36.1).

## Surveillance of *Aedes vexans* in Nottinghamshire, England

As part of long-running UK Health Security Agency (UKHSA) mosquito surveillance to understand nuisance biting (since 2018) near Retford, Nottinghamshire, England [9], mosquitoes were collected using a Mosquito Magnet Executive model (Woodstream Corporation) baited with an octenol lure. Mosquito traps were deployed from July–September 2023 and operational every other week for four nights. A total of 44,080 female mosquitoes were caught in the 2023 trapping season as part of UKHSA surveillance. The total catch was predominately *Ae. vexans* (99.99%); no *Culex pipiens s.l.* or *Cx. modestus* mosquitos were captured. All mosquitoes were morphologically identified using a stereomicroscope.

These mosquitoes were requested for retrospective arboviral screening for the VB-RADAR project in January 2025. Because of laboratory capacity constraints, a subset of 2,000 *Ae. vexans* individuals trapped during the period 11–21 July 2023 was pooled by date of capture into groups of 10 before RNA extraction, undertaken in March 2025. In brief, total nucleic acid was extracted using the KingFisher Flex Purification System (ThermoFisher). Pools that were PCR-positive for WNV were re-extracted using a QIAamp Viral RNA Extraction mini kit (QIAGEN). Additional information regarding mosquito sampling and nucleotide extraction is provided in the appended Supplement. 

### West Nile virus detection and phylogenetic analysis

Nucleotide extracts from all 200 pools of *Ae. vexans* were screened for the presence of WNV RNA, using three PCR assays targeting the 5’-UTR and non-structural gene 5 (NS5) [10-12]. Of these, two *Ae. vexans* pools (hereby referred to as Av_1 and Av_2, both collected on 21 July 2023) tested positive with Cq values ≤ 33.4. Attempts to isolate the virus in Vero cells from the original mosquito homogenate were unsuccessful. The amplicons from positive pools were submitted for Sanger sequencing to confirm their identity. Sequencing produced two amplicons matching the 5’-UTR region and NS5 of WNV lineage 1a (BLAST identity > 95%).

**Figure 2:**
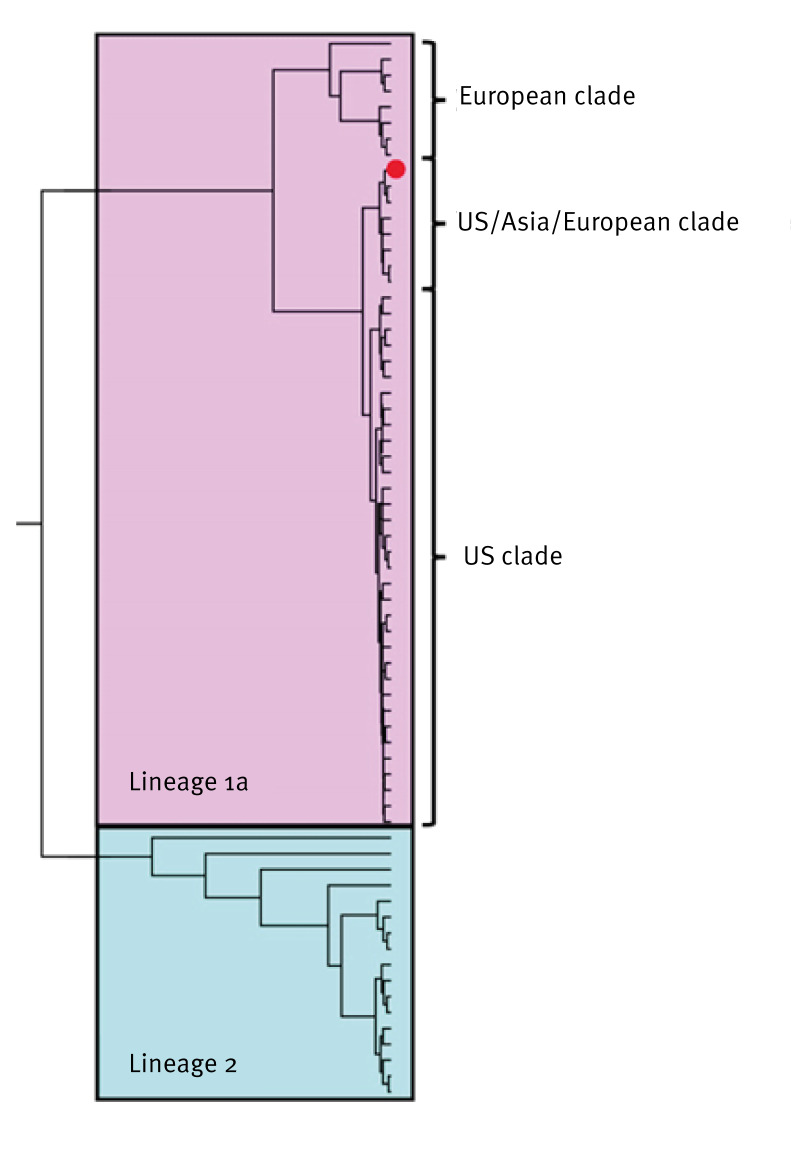
Phylogenetic tree of a 402 bp West Nile virus consensus contig of *Aedes vexans,* United Kingdom, July 2023, aligned against 65 genomes from GenBank

WNV: West Nile virus; UK: United Kingdom; US: United States.

In an attempt to generate additional sequence data, amplicon-based sequencing was undertaken using a GridION (Oxford Nanopore Technologies). Here, a multiplex PCR designed to amplify 37 consecutive sequences of the WNV lineage 1a genome was used (a list of primers used can be found in Supplementary Table S3). The GridION produced ca 140,000 reads for Av_1 and Av_2 combined, over a 15-h sequencing window, all of which were of similar size to the desired 400 bp contigs. Following quality checks and primer removal, no reads mapped to WNV lineage 1a or 2, or to related *Orthoflavivirus* genomes using a combination of Burrows-Wheeler Aligner BWA (v0.7.13) and SAMtools (v1.21). The resulting GridION contig file was converted to a BLAST database and a representative WNV genome (GenBank accession number: OM302321) was used to identify potential matches. Five-hundred and twenty contigs from Av_1 matched the WNV genome (C-terminal of the E gene and N-terminal of NS1); however, no reads from Av_2 matched the WNV template genome. We aligned 52 comparable sequences from Av_1 against a WNV genome in MEGA11 (v11.0.11) to generate a 402 bp consensus sequence for downstream phylogenetic analysis. The *Ae. vexans*-derived WNV consensus sequence was aligned with 65 WNV genomes from GenBank (Figure 2).

Constructed using BEAST (v1.10.4), a Bayesian phylogenetic tree was produced using the GTR + I + G4 nucleotide substitution model and 30,000,000 Markov chain Monte Carlo generations, visualised and annotated in FigTree v1.4.4. The red circle marks the 402 bp United Kingdom sequence (GenBank accession number: PV659664), which clusters within WNV lineage 1a clade. All sequences used in the phylogenetic analysis can be found in Supplementary Table S2.

## Discussion

We present data to support a detection of WNV RNA in mosquitoes collected in Nottinghamshire, England in 2023. Our results from a retrospective analysis in 2025, show that the detection likely represents WNV lineage 1a, which circulates in mainland Europe [13] and the United States (US), where it has a large impact on native bird populations [14]. Despite ongoing surveillance in wild birds and mosquitoes since March 2023 at over 35 field sites across England, we have no evidence of further WNV circulation in the UK to date.

*Aedes vexans* is an uncommon species in the UK, requiring summer flooded wetlands to trigger egg hatching and larval development, and it is only known to occur in a few localised areas of the UK [9,15]. Although not considered a principal enzootic WNV vector in Europe, in contrast to *Culex pipiens *s.s. or *Cx. torrentium* and *Cx. modestus*, *Ae. vexans* is an opportunistic feeder which can occur in high densities and feeds on birds, horses and humans, indicating it could act as a bridge vector [16]. Furthermore, it is a competent vector of several arboviruses including WNV [17]. Consequently, *Ae. vexans* may play an important role in arboviral circulation, particularly where it is abundant, and therefore should be considered in surveillance efforts to appraise risks to public health. However, no evidence exists that this species has been involved in active transmission of WNV in the UK. Meanwhile, local health authorities have been notified of the detection, so that cases of unexplained encephalitis can be fully investigated.

Following high levels of nuisance biting of people at the index site over at least 7 years [9,15], targeted biocidal larviciding by local pest control operators and subsequent land management undertaken by the landowner in conjunction with a local conservation organisation in early 2025, has modified much of the habitat to markedly reduce the area of floodplain in an attempt to reduce densities of *Ae. vexans*. The level of nuisance biting influenced the trapping methods deployed at the site in 2023, specifically targeting *Ae. vexans*. However, ongoing mosquito and arboviral surveillance at the index site, which began in May 2025, utilises cold chain logistics and a range of trapping methods designed to ensure the inclusion of *Culex* vector species for surveillance and improve our ability to detect emerging arboviruses.

Phylogenetic analysis of the UK WNV detection indicates a close evolutionary relationship to WNV lineage 1a sequences detected in the US, the Middle East and Europe. However, this is based on a short, conserved region of the WNV genome and, consequently, we are unable to determine the geographic origin of this incursion [18]. Although the *Orthoflavivirus* NS5 coding sequence is conserved, contigs generated from Sanger sequencing did not map to related viruses, such as USUV, providing high confidence in the detection of WNV in two historic samples.

While WNV incursion into the UK is likely to have occurred through either long- or short-distance bird migration from mainland Europe, natural- or human-facilitated mosquito movement cannot be discounted [19]. Human-facilitated mosquito movement has been implicated as a possible incursion method of WNV into North America in 1999 [14], and has been highlighted as a means of incursion into other island ecosystems [19]. Although the origin of this incursion into the UK remains uncertain, current climatic conditions influencing the extrinsic incubation period suggest that WNV would not be able to circulate sustainably at the index site [20].

## Conclusions

While our detection of WNV RNA in a potential mosquito bridge vector indicates that the UK is permissive for incursion events, the public health risk of WNV is currently considered very low. However, following heat waves across mainland Europe and the UK in June 2025, climatic conditions are similar to those experienced in June 2023. Although current models suggest that the climate would inhibit WNV establishment in the UK, prolonged heat waves could promote viral replication increasing the possibility of irregular outbreaks and emphasising the need for continued arbovirus surveillance across the UK. We advocate that an integrated approach to surveillance, involving vectors, wildlife, domestic animals and people, is essential to rapidly identify the temporo-spatial presence of infection and to mitigate transmission to humans.

## Data Availability

The sequence for the UK West Nile virus lineage 1a detection used for analysis can be found at GenBank under accession number: PV659664.

## Supplementary Data

254000 Supplement
