# Supplementary material for: Detection of West Nile virus via retrospective mosquito arbovirus surveillance, United Kingdom, 2025
Source: Euro Surveill. 2025 Jul 17;30(28):2500401. doi: 10.2807/1560-7917.ES.2025.30.28.2500401 (PMC12273253; doi:10.2807/1560-7917.ES.2025.30.28.2500401)
Supplement: Supplement [file 25-00401_BRUCE_Supplement.pdf]

This supplementary material is hosted by Eurosurveillance as supporting information alongside the article "Detection of West Nile virus via retrospective mosquito arbovirus surveillance, United Kingdom, 2023" on behalf of the authors who remain responsible for the accuracy and appropriateness of the content. The same standards for ethics, copyright, attributions and permissions as for the article apply. Eurosurveillance is not responsible for the maintenance of any links or email addresses provided therein.

Supplementary Methodology

Mosquito sampling at Gamston

Of the 31,582 mosquitoes collected and screened for WNV RNA as part of the Vector-Borne RADAR project, the 2,000 female *Ae. vexans* from Gamston were selected due to their vectoral-capacity for Tahyna virus [19]. Due to laboratory capacity restraints, it was not possible to process the full collection of 44,080 mosquitoes (of which 99.99% were *Ae.vexans*) from Gamston in this project. Whilst additional *Ae. vexans* samples were collected from a second site in Suffolk 2024 (Supplementary Table 1), it was not possible to collect samples at Gamston due to construction works (see main text).

The authors note that the primary vectors of WNV (specifically *Cx. pipiens s.s.* and *Cx. modestus*) were not trapped at this site in 2023 for two reasons. Firstly, the octenol lure used during trapping is specific for mammalophagic species and thus would not attract the ornithophagic *Cx. pipiens pipiens.* Secondly, *Cx. modestus* and *Cx. p. molestus* are only found in localised areas of the UK and have not been recorded at the Gamston site to date [37].

Nucleotide extraction

Mosquito pools were homogenised in 500 µl of MagMAX™ CORE Lysis Solution (Thermo Fisher, UK), using a 5 mm steel bead and a TissueLyser2 (Thermo Fisher, UK) for 3 minutes at 30 rps. Initial nucleotide extraction was then conducted using 350 µl of this lysate and a KingFisher Flex Purification System (Thermo Fisher, UK) following the manufacturer's instructions and eluted in 50 µl of MagMAX™ CORE elusion buffer (Thermo Fisher, UK).

Where WNV RNA was detected, viral RNA was re-extracted from 150 µl lysate utilising a QIAamp Viral RNA Extraction mini kit (QIAGEN, UK) following the manufacturer's instructions and eluted in 50 µl of Buffer AVL (QIAGEN, UK).

WNV Multiplex PCR

Positive sample pools were subjected to a multiplex PCR to amplify consecutive 400 bp regions of the WNV lineage 1a genome. Reverse transcription was conducted on viral RNA extracts using LunaScript© (New England Biolabs, USA) according to the manufacturer's instructions. Primers obtained from PrimalScheme (yale-west-nile-virus/400/v1.0.0) [38], were separated into two pools (as indicated in Supplementary Table 3). PCR reactions comprised 12.5 µl Q5® High-Fidelity 2x master mix (New England Biolabs, USA), 4 µl of respective primer pool (1 or 2), 2.5 µl of synthesised cDNA and 6 µl of molecular grade water.

Reactions were subjected to conventional PCR using the following cycling conditions: initial denaturation at 98 ºC for 30 seconds; 35 cycles of 98 ºC for 10 seconds, 62 ºC for 30 seconds and 72 ºC for 30 seconds, and a final 5-minute extension period at 72 ºC.

Supplementary Table S1: The total number (*n*) of individual mosquitoes caught between March 2023-March 2025, inclusive, and analysed as part of the Vector-Borne RADAR project. Samples were pooled (≤13) by species, sex, location and collection date. All samples tested negative for WNV RNA, except two pools of the 2000 *Aedes vexans* (marked in **bold**). Where possible mosquito specimens were kept at -20 ºC immediately following capture, all samples were transferred to -80 ºC for long-term storage. * indicates specimens identified to genus level but unidentifiable at species level.

	March-October 2023		January-November 2024		January-March 2025			
Mosquito species	Total Female (*n*)	Total Male (*n*)	Total Female (*n*)	Total Male (*n*)	Total Female (*n*)	Total Male (*n*)	Total individuals (*n*)	Total number of pools (*n*)
Aedes cantans/annulipes	901	1	1169	0	0	0	2071	252
Aedes caspius	250	0	86	0	0	0	336	48
Aedes cinereus/geminus	102	0	2298	0	0	0	2400	260
Aedes communis	0	0	8	0	0	0	8	3
Aedes detritus	1854	0	1011	0	0	0	2865	323
Aedes dorsalis	0	0	3	0	0	0	3	2
Aedes flavescens	374	0	59	0	0	0	433	49
Aedes geniculatus	4	1	1	0	0	0	6	4
Aedes leucomelas	0	0	1	0	0	0	1	1
Aedes punctor	0	0	4	0	0	0	4	3
Aedes rusticus	673	0	51	0	0	0	724	89
Aedes sticticus	0	0	2	0	0	0	2	2
Aedes vexans	**2000**	0	1380	0	0	0	3380	338
*Aedes spp.**	1	0	12	0	0	0	13	4
Anopheles claviger	3229	15	815	1	0	0	4060	453
Anopheles maculipennis s.l.	51	4	44	1	0	0	100	34
Anopheles plumbeus	58	0	89	0	0	0	147	38
Coquillettidia richardii	884	0	1438	0	0	0	2322	285
Culex modestus	1026	0	1907	0	0	0	2933	311
Culex pipiens s.l.	6522	7	949	2	514	0	7994	859
*Culex spp.**	14	0	27	0	0	0	41	8
Culiseta annulata	949	10	318	0	5	0	1282	206
Culiseta morsitans	435	0	16	0	0	0	451	65
Unknown	0	0	6	0	0	0	6	2
Total	19327	38	11694	4	519	0	31582	3639

Supplementary Table S2: The sixty-five West Nile virus sequences obtained from GenBank used in phylogenetic analysis. Sequences were selected from the available Genbank repository with a broad temporal and spatial representation (six from Africa (1958-2006); two from Asia (1998-2005); 18 from Europe (2916-2018) and 38 from North America (1999-2022) to provide coverage of the West Nile virus evolutionary history.

Accession number	Date	Location Country	Continent	Lineage
HM147824	1958	Democratic Republic of Congo	Africa	L2
HM147822	1958	South Africa	Africa	L2
HM147823	1988	Madagascar	Africa	L2
OP870459	1998	Senegal	Africa	L1a
OP870461	2006	Senegal	Africa	L1a
ON813219	2006	Senegal	Africa	L2
AF481864	1998	Israel	Asia	L1a
AY688948	2005	Israel	Asia	L2
OM302316	2016	Spain	Europe	L1a
OM302319	2017	Spain	Europe	L1a
OM302317	2018	Spain	Europe	L1a
MH021189	2018	Belgium	Europe	L2
MH924836	2018	Germany	Europe	L2
OP713602	2020	Spain	Europe	L1a
OP713603	2020	Spain	Europe	L1a
MW036634	2020	Netherlands	Europe	L2
OP762597	2020	Netherlands	Europe	L2
MW036633	2020	Netherlands	Europe	L2
MW036634	2020	Netherlands	Europe	L2
OP609793	2022	Italy	Europe	L1a
OP609798	2022	Italy	Europe	L1a
OP609813	2022	Italy	Europe	L1a
PQ450190	2024	Poland	Europe	L2
PQ483500	2024	Spain	Europe	L2
PQ481864	2024	Spain	Europe	L2
MN619803	2018	Russia	Europe	L2
NC009942	1999	New York, USA	North America	L1a
AF404756	2000	New York, USA	North America	L1a
KJ786934	2001	New York, USA	North America	L1a
HQ671697	2001	USA	North America	L1a
KX547194	2002	New York, USA	North America	L1a
KX547287	2003	New York, USA	North America	L1a
JQ700437	2003	Ney York, USA	North America	L1a
HM488145	2004	USA	North America	L1a
MH819448	2005	Canada	North America	L1a
OQ721162	2005	Minnesota, USA	North America	L1a
JF415914	2005	Texas, USA	North America	L1a
MH819469	2006	Canada	North America	L1a
JF415916	2006	Texas, USA	North America	L1a
JF415915	2006	Texas, USA	North America	L1a
JF957171	2007	Indiana, USA	North America	L1a
JF415918	2007	Texas, USA	North America	L1a
JF957170	2007	USA	North America	L1a
JF957173	2008	Arizona, USA	North America	L1a

JN183886	2008	New York, USA	North America	L1a
JF415927	2008	Texas, USA	North America	L1a
JF957176	2009	Arizona, USA	North America	L1a
OQ721116	2009	California, USA	North America	L1a
JF488095	2009	New York, USA	North America	L1a
JF957183	2009	Texas, USA	North America	L1a
JQ700438	2011	USA	North America	L1a
KY216149	2012	New York, USA	North America	L1a
MH819447	2013	Canada	North America	L1a
OK631659	2013	New York, USA	North America	L1a
MH819446	2015	Canada	North America	L1a
OK631660	2015	New York, USA	North America	L1a
MT967990	2015	New York, USA	North America	L1a
OQ721131	2016	California, USA	North America	L1a
MT967996	2017	New York, USA	North America	L1a
OQ721122	2018	Illinois, USA	North America	L1a
MT968010	2018	New York, USA	North America	L1a
OQ721111	2018	Ohio, USA	North America	L1a
MZ595325	2021	New York, USA	North America	L1a
MZ595324	2022	New York, USA	North America	L1a

Supplementary Table S3: Primers used in the multiplex primer tiling PCR, available at PrimalScheme [38]. Pools 1 and 2 were run in separate PCR reactions and the products combined prior to barcoding during GridION sample preparation.

**Forward**	**Reverse**	**Pool**
GCCTGTGTGAGCTGACAAACTT	GATAGCACTGGTCAAGGTCCCT	1
GCGTTCTTCAGGTTCACAGCAA	GACTTTGTGCACCAACAGTCGA	2
TGTCAGAGCAATGGATGTGGGA	CTGTTGCTCATTCCAAGGCAGT	1
TCATTGGTTGGATGCTTGGGAG	TGTGTCAATGCTTCCTTTGCCA	2
AATGACAAACGTGCTGACCCAG	CACTCACGATGGACCAAGAACG	1
ATATGGAGAGGTGACAGTGGACT	AAAGCCTTTGAACAGACGCCAT	2
TTCAAGCAACACTGTCAAGTTAAC	CTGCTTCCAGACTTGTGCCAAT	1
GCCAACGCTAAGGTCCTGATTG	ACGGAGAGGAAGAGCAGAACTC	2
GCATGTCCTGGATAACGCAAGG	AACCACGACACTAAGGTCCACA	1
TCTACGATCAGTTTCCAGACTGGA	GTTGTTCTTGACAGCCGTTCCA	2
TGGAGGATTTTGGATTTGGTCTCAC	AGTCAATCTCTACCCGGCCTTC	1
GGCGATGGAATCCTTGAGAGTG	GGCCCAACTGAAAAGGGTCAAT	2
CGGCTGTTGGTATGGTATGGAG	TTCTCCTGGTTGGTCCATCTCG	1
CTAATTCGGGAGGAGACGTGGT	CCTGATCAAGCTGCCTATTCCG	2
ATCAAACGTGGTTGTTCCGCTG	CACGAAAGCAGCAAACATGAGC	1
TGTCGGCCTGATGTTTGCCATC	GGTCGTGTCCCCCTTTTTGTAC	2
TGCCCTCAGTAGTTGGATTTTGG	TTTGAACACCCCTGGTTTCGTC	1
TGGAAATTGCAGCACAAGTGGA	GGCCTCTTTGATGATCTGTGGC	2
CGGATTCGAACCTGAGATGCTG	AACCTCTTGCTGCAATGCTAGC	1
ACAGGCTGATGTCTCCTCACAG	ACATTTTGGGTACTCCGTCTCGT	2
GTGCCTAGTGTCAAGATGGGGA	GATTCGTGCCTCAGTCCAATGG	1
CGGTAGAAATCCGTCGCAAGTT	GCGGCCTCAGAATCTTCCTTTC	2
AGGTGGTGCTTTGATGGTCCTA	ACTCCCATGGTCATCACACTCA	1
TGCAGAGAAAGGAGGAAGAGCT	CCTTGACCTCAATTCTTTGCCCA	2
TTGTGTCATGACCCTTGTGAGC	GGAACCATGTAGGCATAGTGGC	1
CTTCGTCGATGTTGGAGTGTCG	CTCCATTCTCCCAAAGCGTCAC	2
TCTTGGTGTCTCTAGCTGCAGT	CAGTTTTGCTGTGCCCCTAGAG	1
GTACCGCAAAGAGGCCATCATC	TTGACGAGGACTCTCCGATGTC	2
TGGAACATTGTCACCATGAAGAGT	CTTCCTCGTATTGGGGTCCCTT	1
CGAGCTTCAGGCAATGTGGTAC	AGGGAGTAGTGTCAGTCATGGC	2
CAGTTCGCTGGTCAATGGAGTG	AATGCAAGTGTGACATTCCCCC	1
GAGCAGAATCAATGGAGGAGCG	TCCAAGTCAGCTCTCGTGATGC	2
TTACATCCTGCGTGAAGTTGGC	TCTCCACATCATCTGGGCCAAT	1
TGTCACCTACGCCCTAAACACT	GGAGATATGCGAGCTCTGCCTA	2
CAGGTTCCATTTTGCTCAAACCA	TCACTCCATTTCTCCACTGGGG	1
CATGCAGGAGGAGAGTGGATGA	CGGCCTGACTTTCTCCTCAAAA	2
TGGTTGAGGACACAGTACTGTAGA	GTCCTTTCGCCCTGGTTAACAC	1
GCGAAGTGATCCATGTAAGCCT	TGTGCAGAGCAGAAGATCTCCT	2
